# Human Dynactin-Associated Protein Transforms NIH3T3 Cells to Generate Highly Vascularized Tumors with Weak Cell-Cell Interaction

**DOI:** 10.1371/journal.pone.0135836

**Published:** 2015-08-18

**Authors:** Tatsuki Kunoh, Weixiang Wang, Hiroaki Kobayashi, Daisuke Matsuzaki, Yuki Togo, Masahiro Tokuyama, Miho Hosoi, Koichi Koseki, Shu-ichi Wada, Nobuo Nagai, Toshinobu Nakamura, Shintaro Nomura, Makoto Hasegawa, Ryuzo Sasaki, Tamio Mizukami

**Affiliations:** 1 Graduate School of Bioscience, Nagahama Institute of Bio-Science and Technology, Nagahama, Shiga, Japan; 2 Frontier Pharma, Nagahama, Shiga, Japan; Boston University Goldman School of Dental Medicine, UNITED STATES

## Abstract

Human dynactin-associated protein (dynAP) is a transmembrane protein that promotes AktSer473 phosphorylation. Here, we report the oncogenic properties of dynAP. In contrast to control NIH3T3 cells expressing LacZ (NIH3T3LacZ), NIH3T3dynAP cells vigorously formed foci in two-dimensional culture, colonies on soft agar, and spheroids in anchorage-deficient three-dimensional culture. NIH3T3dynAP cells injected into nude mice produced tumors with abundant blood vessels and weak cell—cell contacts. Expression of dynAP elevated the level of rictor (an essential subunit of mTORC2) and promoted phosphorylation of FOXO3aSer253. FOXO3a is a transcriptional factor that stimulates expression of pro-apoptotic genes and phosphorylation of FOXO3a abrogates its function, resulting in promoted cell survival. Knockdown of rictor in NIH3T3dynAP cells reduced AktSer473 phosphorylation and formation of foci, colony in soft agar and spheroid, indicating that dynAP-induced activation of the mTORC2/AktSer473 pathway for cell survival contributes to cell transformation. E-cadherin and its mRNA were markedly reduced upon expression of dynAP, giving rise to cells with higher motility, which may be responsible for the weak cell-cell adhesion in tumors. Thus, dynAP could be a new oncoprotein and a target for cancer therapy.

## Introduction

The PI3K-Akt-mammalian (officially mechanistic) target of rapamycin complex (mTORC) signaling pathway plays critical roles in the regulation of a wide range of cellular processes including growth, proliferation, and survival [1–6]. Deregulated activation of this pathway has been implicated in a number of pathological conditions including cancer [6]. mTORC is a large serine (Ser)/threonine (Thr) kinase complex that exists in mammals as two types of complexes (mTORC1 and 2). Rapamycin-sensitive mTORC1 consists of mTOR, raptor, and other subunits, while rapamycin-insensitive mTORC2 consists of mTOR, rictor, and other subunits. Growth factor receptors activated by binding of ligands activate PI3K, increasing production of PI(3,4,5)P3. Akt binds to this phospholipid at the plasma membrane where phosphatidylinositol-dependent protein kinase (PDK1/PDPK1) phosphorylates Thr308 in the activation loop of Akt. This phosphorylation results in partial Akt activation, but it is sufficient to activate the route to mTORCl. Activated mTORC1 phosphorylates eukaryotic translation initiation factor 4E binding protein 1 (4E-BP1) and ribosomal protein S6 kinase, 70 kDa, polypeptide 1 (S6K), promoting protein synthesis as well as cell growth and proliferation. In addition, Akt is phosphorylated at Ser473 in the C-terminal hydrophobic motif, which produces Akt with higher activity and altered substrate specificity. mTORC2 [7] and DNA-dependent protein kinase (DNA-PK) [8] have been shown to phosphorylate AktSer473. The presence of the rictor subunit in mTORC2 appears to dictate the substrate specificity of mTOR towards AktSer473. Akt phosphorylated at Ser473 acquires the capability to phosphorylate additional substrates including FOXO transcriptional factors that promote expression of pro-apoptotic genes [9, 10]. Phosphorylation of FOXO proteins inhibits their nuclear translocation, thereby supporting cell survival.

Previously, we reported that the human C18orf26 gene encodes a protein that is expressed in half of the tested human cancer cell lines but barley in normal cells [11]. This protein was designated as dynAP (dynactin-associating protein) because of its interaction with dynactin subunits that compose a microtubule-based motor protein complex. DynAP is a transmembrane protein localized to the Golgi apparatus and plasma membrane. Overexpression of dynAP in HeLa cells promotes phosphorylation of Akt at Ser473, whereas knockdown of endogenous HeLa dynAP abolishes basal phosphorylation of AktSer473. Although the physiological function(s) of dynAP are unknown, these observations suggest that dynAP may be oncogenic. In this study, we demonstrate dynAP-induced oncogenic transformation of mouse cells. This study also shows that dynAP-induced upregulation of rictor, an essential subunit of mTORC2, is critical for cell transformation.

## Materials and Methods

### Cells and cultures

Parental NIH3T3 cells expressing EGFP and NIH3T3H-Ras cells (NIH3T3 cells expressing EGFP and mutant H-RasG12V) were maintained in Dulbecco's modified Eagle's medium (DMEM) supplemented with 4.5g/l glucose (Nacalai Tesque, Kyoto, Japan) and 10% fetal calf serum (FCS) (JRH Biosciences, St. Louis, MO, USA). The human cell lines and media used in this study have been described previously [11].

### Preparation of EGFP- and H-Ras-expressing NIH3T3 cells

pMY-IRES-EGFP or pMY-H-Ras-IRES-EGFP retroviral vectors were introduced into Plat-E cells using FuGENE 6 transfection reagent (Roche, Indianapolis, IND, USA) according to the manufacturer’s recommendations. After 48 hours, virus-containing supernatants were filtered through 0.45-μm cellulose acetate filters and supplemented with 8 μg/ml polybrene (Sigma-Aldrich, St.Louis, MO, USA). Target cells were then incubated overnight with the virus/polybrene-containing supernatants. After infection of the cells, the medium was replaced with fresh medium.

### Lentivirus-mediated expression of dynAP

Full-length dynAP cDNA (NCBI accession number: NM_173629.1) was cloned into the pLenti6.3/V5-DEST vector (Life Technologies, Carsbad, CA, USA). The pLenti6.3/V5-GW/LacZ vector was used as a control. Production of the virus and infection of mammalian cells to express dynAP or LacZ were performed according to the manufacturer’s protocol. Briefly, lentiviruses were generated in HEK293FT cells by co-transfection of pLenti vectors and ViraPower Lentiviral Packaging Mix (Life Technologies). The supernatants were harvested at 48 hours after transfection and used to infect NIH3T3 and NIH3T3H-Ras cells. Infected cells were selected with 5 μg/ml blasticidin S (Sigma-Aldrich) for half a month. Drug-resistant cells were pooled to rule out clonal artifacts. The following random clones were used in this study: NIH3T3LacZ (as a control), NIH3T3dynAP, NIH3T3H-Ras and NIH3T3H-RasdynAP cells.

### RNA interference analyses

Short hairpin RNA (shRNA)-mediated knockdown of rictor in NIH3T3dynAP cells was performed according to the manufacturer’s protocol (Life Technologies). DNA oligonucleotide sequences used to generate pre-miRNA sequences were designed by RNAi designer (Life Technologies). Two oligonucleotides (“top strand”) were designed for knockdown of mouse rictor, rictor_shRNA494 (5’-TGCTGAGAACTAGGAAACAAGGAAGCGTTTTGGCCACTGACTGACGCTTCCTTTTCCTAGTTCT-3’) and rictor_shRNA2783 (5’-TGCTGAAGAGAGGCTTTCAGCTTCTTGTTTTGGCCACTGACTGACAAGAAGCTAAGCCTCTCTT-3’). shRNA numbers indicate nucleotide numbers from the 5’ end of mouse rictor mRNA (NCBI accession number: NM_030168.3) and the underlined 21 antisense target sequences start at these positions. For more efficient knockdown of rictor, we also used the oligonucleotide rictor-shRNA494_2783 in which rictor_shRNA494 and rictor_shRNA2783 were connected in tandem. After annealing these oligonucleotides to complementary oligonucleotides, the double-stranded DNAs were cloned into the pcDNA6.2-GW/EmGFP-miR vector. The blasticidin resistance gene in this vector was replaced with a neomycin resistance gene, because NIH3T3 cells expressing dynAP already harbored a blasticidin resistance gene. The resulting primary shRNA expression plasmids were introduced into NIH3T3dynAP cells using Lipofectamine 2000 (Life Technologies). As a negative shRNA control, we used the pcDNA6.2-GW/EmGFP-miR-neg control plasmid that produces a primary shRNA transcript with a hairpin structure for processing, but the resulting shRNA is predicted not to target any known vertebrate genes (Life Technologies). The transfected cells were cultured in the presence of 5 μg/ml blasticidin and 800 μg/ml G418 (Funakoshi, Tokyo, Japan).

### Preparation of cell extracts and immunoblotting

Harvested cells were washed once with PBS and lysed in Laemmli’s sample buffer. Cell lysates were incubated on ice for 10 minutes and then at 95°C for 10 minutes. The lysates were clarified by centrifugation at 16,000×*g* for 5 minutes, and the supernatants were collected. The protein concentrations were determined with a Protein Assay Kit (Bio-Rad Laboratories, Hercules, CA, USA) using bovine gamma-globulin as a standard. The supernatants were subjected to 10% SDS-polyacrylamide gel electrophoresis. The separated proteins were transferred to nitrocellulose membranes (Pall Corporation, Port Washington, NY, USA). The membranes were then incubated with the following primary antibodies against mouse antigens. Antibodies were purchased from indicated sources: anti-Akt, anti-phospho-AktSer47, anti-S6K, anti-sphopho-S6KThr389, anti-4E-BP1, and anti-FOXO3a from Abcam (Cambridge, MA, USA); anti-phospho-4E-BP1Ser65, and anti-phospho-FOXO3Ser253 from Cell Signaling Technology (Danvers, MA, USA); anti-p53, anti-rictor, anti-β-actin, and anti-E-cadherin from Santa Cruz Biotechnology (Santa Cruz, CA, USA). The anti-dynAP antibody was prepared as described previously [11]. A goat anti-rabbit IgG conjugated to horseradish peroxidase (MBL, Nagoya, Japan) was used as a secondary antibody.

Chemiluminescent signals from immunoreactive proteins were produced with Chemi-Lumi One (Nacalai Tesque) and captured using a luminescent image analyzer (LAS-4000; GE Healthcare Japan, Tokyo, Japan). The relative intensities of the bands were quantified using ImageQuantTL software.

### RT-PCR

RT-PCR was performed as described previously [11]. Briefly, total RNA was extracted using an RNeasy kit (Qiagen, Chatsworth, CA, USA) according to the manufacturer's protocol. cDNA was synthesized from 1 μg total RNA using reverse transcriptase and random primers (Toyobo, Osaka, Japan). cDNAs were amplified by PCR using primer sequences for mouse genes as follows: rictor FWD, 5’- AACGTCCCGCTCGATCTGA and REV, 5’- CTTTCTGACTAAGCGAAGGGC; and REV, 5’-TCTGAGCCTTCAGGACCATAGAC; E-cadherin FWD, 5’-GACGATGACGTCAACACCTACAAC and REV, 5’- ATGTAGGGTAACTCTCTCGGTCCAG; β-actin FWD, 5’- GTTCTACAAATGTGGCTGAGGA and REV, 5’- ATTGGTCTCAAGTCAGTGTACAG. Primers for detection of G3PDH mRNA were included in the ReverTraAceAlpha Kit (Toyobo). Primers for detection of dynAP mRNA were as follows: FWD, 5’-GAAGATCTAGTTGCAGATATATAAAGGGCAATG-3’ and REV, 5’-ACGCGTCGACTTATAAATGATCGGTAGGTG. The relative intensities of the bands were quantified using ImageQuantTL software.

### Cell proliferation assay

Cells were seeded in a 96-well tissue culture plate at 1 × 10^4^ to 5 × 10^4^ cells/ml and cultured. The cultures were thoroughly mixed with 10 μl of 5 mg/ml MTT (Sigma-Aldrich) per well and incubated to allow metabolization of the MTT. After 3 hours, the medium was discarded and formazan, a MTT metabolic product, was dissolved in 100 μl lysis buffer (0.4 M HCl in 2-propanol). Then, optical densities were measured at 570 nm and background subtracted at 750 nm.

### Focus formation assay

Cells were seeded in a tissue culture plate and cultured for the indicated periods. Then, the cells were fixed in ice-cold methanol and stained with a 0.5% crystal violet (Nacalai Tesque) solution to identify the presence of cell colonies.

### Wound healing assay

A wound-healing assay was performed to evaluate cell migration. Cells were seeded in a 6-well tissue culture plate at 4 × 10^5^ cells/ml. After overnight culture, a scratch wound was introduced to the cell monolayer with a pipette tip. The plates were washed twice with medium to remove detached cells. The cultures were continued for another 14–18 hours. Images at the start and end points of the cultures were obtained to estimate the cell migration to close the wound.

### Soft agar assay

Soft agar assays were performed in cell growth medium (DMEM plus 10% FCS) containing 100 U/ml penicillin and 100 μg/ml streptomycin using 6-cm dishes (Techno Plastic Products AG, Schaffhausen, Switzerland) in triplicate. For each dish, 2 × 10^4^ cells were mixed thoroughly with 0.3% agar (#01028–85; Nacalai Tesque), and the mixture (2 ml) was plated onto a layer of 0.6% agar (4 ml). Then, 4 ml of 0.6% agar was plated as the top layer. After culture for the indicated periods, colonies were counted and photographed.

### 3D culture with ultralow attachment plates

For spheroid formation, cells were seeded at a density of 500 cells in 100 μl of cell growth medium per well on a U-bottom 96-well ultralow cell adhesion plate (PrimeSurface 96U plate P/N MS-9096U from Sumitomo Bakelite, Tokyo, Japan). The well shape promoted the formation of single, centrally located spheroids of a reproducible size. Spheroid growth was followed by measuring the pseudo-volume (area × optical density) with Cell^3^iMager scanner (SCREEN Holdings, Kyoto, Japan).

### Flow cytometry

To analyze apoptotic cells in 3D culture, flow cytometry was conducted as described previously [11].

### Graft experiments

Animal experiments were performed in strict accordance with the recommendation in the Guide for the Care and Use of Laboratory animals of Japan Society for the Promotion of Science. The protocol was approved by the Committee on the Ethics of Animal Experiments of the Nagahama Institute of Bio-Science and Technology (Permit No. 037). Ten-week-old female nude mice (BALB/cAJcl-nu/nu) were purchased from CLEA Japan, Tokyo, Japan. Cells were washed with PBS twice and resuspended in PBS. An aliquot of 100 μl containing 3 × 10^5^ NIH3T3 cells was injected subcutaneously into the right flank of nude mice using a 29 G needle. Symptom criteria, e.g. behavioral or movement abnormality, hyperalgesia, and decrease in food intake were monitored in every two days to evaluate animal health and suffering. Tumor size was monitored in every two or three days, in which long and short diameter were measured. The endpoint to euthanize animals was set as a tumor burden greater than 10% body weight. Therefore, a tumor should not exceed 20 mm in any one dimension in mice. For pain relief, anesthetic was used for euthanasia by intraperitoneal administration of 200mg/kg sodium pentobarbital. The maximum tumor was seen in NIH3T3H-RasdynAP cells-engrafted tumor group, and the size and weight were measured as 9 mm x 7 mm and 0.93g, respectively. The tumor volume was not measured. The skin surface of maximum tumor in NIH3T3H-RasdynAP cells-engrafted tumor group appeared to be partly ulcerated with redness, whereas in NIH3T3H-Ras cells-engrafted tumor group appeared to be partly blistered with slight redness. The blistering lesions were evaluated by the sizes and degrees of redness, blister or ulcer, and the symptoms and signs associated with itching and scratching with respect to the degree of inflammation, oedema or erythema. Since no symptoms and signs associated with itching and scratching were observed, we evaluated that these skin lesions did not cause severe suffering for the tumor bearing mice. Animal weight changes during the course of the experimental period were monitored. The weights of tumor-bearing mice increased greater than did control mice during the course of graft experiments, conceivably reflecting tumor burden, indicating normal weight gains in tumor-bearing mice. No abnormal vital signs, including weight loss, were seen for the tumor bearing mice as well as control mice.

### Histological analyses

Tumors were removed from total 27 mice (nine mice for NIH3T3dynAP cells, nine for NIH3T3H-RasdynAP cells, and nine for NIH3T3H-Ras cells), fixed in 10% formalin, sectioned, and embedded in paraffin. The paraffin-embedded specimens were sectioned at 5 μm thicknesses. The sections were stained with hematoxylin and eosin. To evaluate blood vessel formation in a quantitative manner, the sections were subjected to immunohistochemical analysis using an antibody against CD34. For antigen retrieval, deparaffinized sections were boiled in Target Retrieval Solution (Dako Japan, Tokyo, Japan) at 120°C for 20 minutes. The sections were incubated with 0.5% (v/v) H_2_O_2_ in methanol for 30 minutes to eliminate endogenous peroxidase activity and then treated with blocking reagent (PerkinElmer, Waltham, MA, USA). Then, the sections were incubated for 1 hour at room temperature with a primary antibody against rat CD34 (Abcam). After washing with Tris-NaCl buffer containing 0.05% Tween 20, the sections were incubated with a peroxidase-conjugated secondary antibody (Abcam) for 1 hour. Peroxidase activity was visualized with 3,3’-diaminobenzidine. Blood vessels were evaluated by measuring the CD34-positive area or counting the CD-34-positive number using NIH ImageJ software. Quantification of blood vessels was performed using two randomly selected sections for each mouse.

### Statistical analysis

Data except those of allograft experiments were analyzed by the Student’s t-test with Welch’s correction. A *p* < 0.05 was considered statistically significant. Data of allograft experiments were analyzed by the Mann-Whitney test.

## Results

### Expression of human dynAP transforms NIH3T3 cells

**Fig 1:**
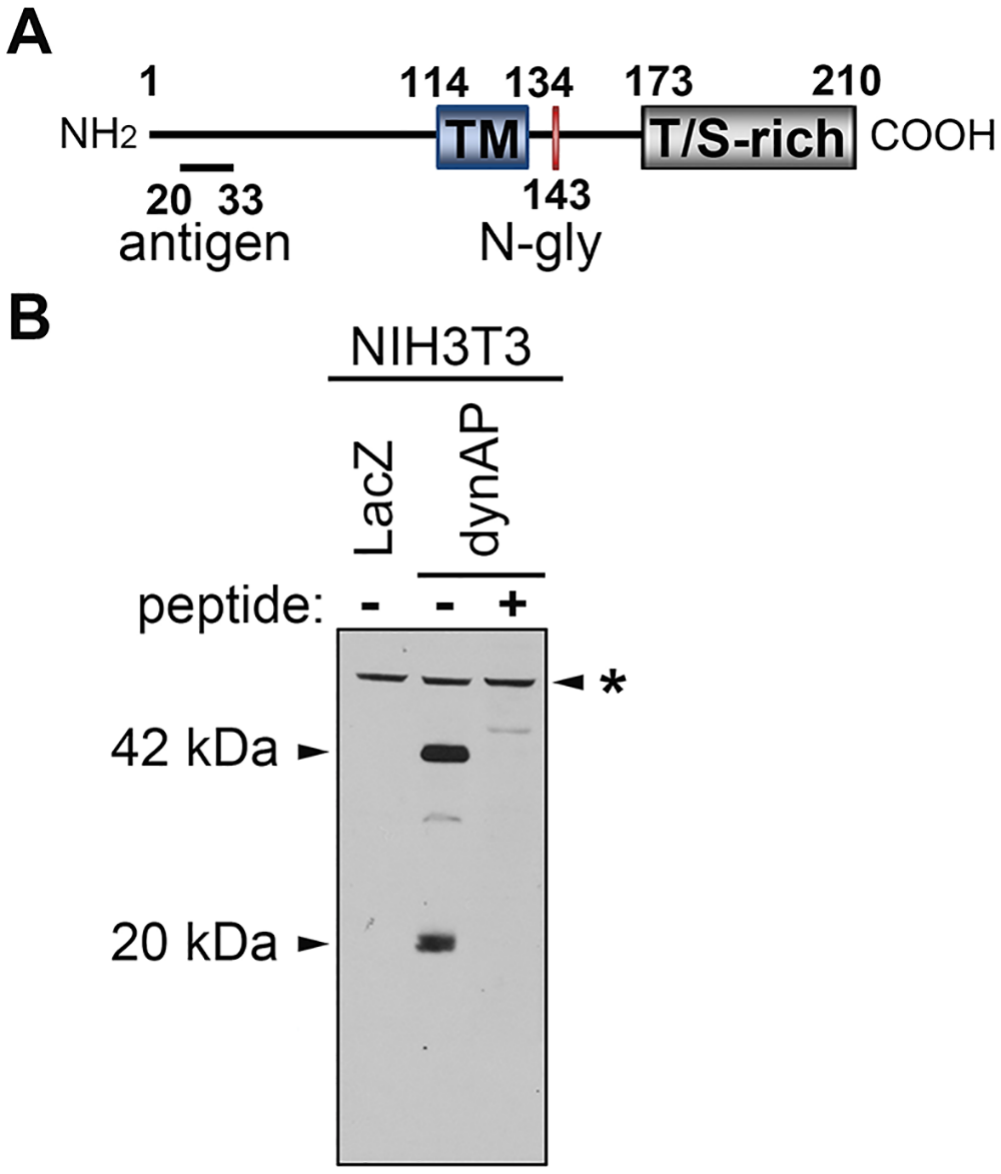
Gross structure of human dynAP and its expression in NIH3T3 cells. (A) Structure of dynAP showing the transmembrane domain (TM), N-glycosylation site (N-gly), threonine/serine rich region (T/S-rich), and the antigen peptide (aa. 20–33) used for raising the rabbit antibody. (B) Immunoblot analyses of dynAP expressed in NIH3T3 cells. Lane 1, NIH3T3LacZ cell lysate and lane 2, NIH3T3dynAP cell lysate. In lane 3, the NIH3T3dynAP cell lysate was analyzed with an antibody that had been pretreated with 10 nM of the antigen peptide at 37°C for 30 minutes. *non-specific.

Human dynAP is a type II transmembrane protein consisting of 210 amino acids with its C-terminal region exposed to the outside of cells (Fig 1A) [11]. The C-terminal region contains a possible N-glycosylation site and many Thr/Ser residues (20/38 residues) that may undergo O-linked glycosylation. Parental NIH3T3 cells expressing enhanced green fluorescent protein (EGFP) were engineered to express dynAP or LacZ (as a control) and cultured in the presence of blasticidin. Hereafter, we refer to NIH3T3 random clones expressing dynAP or LacZ as NIH3T3dynAP and NIH3T3LacZ cells, respectively. DynAP expression was examined using an antibody against a dynAP peptide (amino acids 20–33 in Fig 1A). A major 42 kDa component and a minor 20 kDa component were detected in NIH3T3dynAP cells but not in control NIH3T3LacZ cells. The interaction of these components with the antibody was completely blocked by pretreatment of the antibody with the antigen peptide (Fig 1B). A molecular mass of 22.5 kDa was calculated from the amino acid sequence, suggesting a modification of 42 kDa dynAP. At present, it is unknown whether glycosylation is fully accountable for the difference in molecular sizes. The minor 20 kDa component in NIH3T3dynAP cells may be an unmodified form or degradation product. Morphological differences were not found among parental NIH3T3, NIH3T3LacZ and NIH3T3dynAP cells (data not shown).

**Fig 2:**
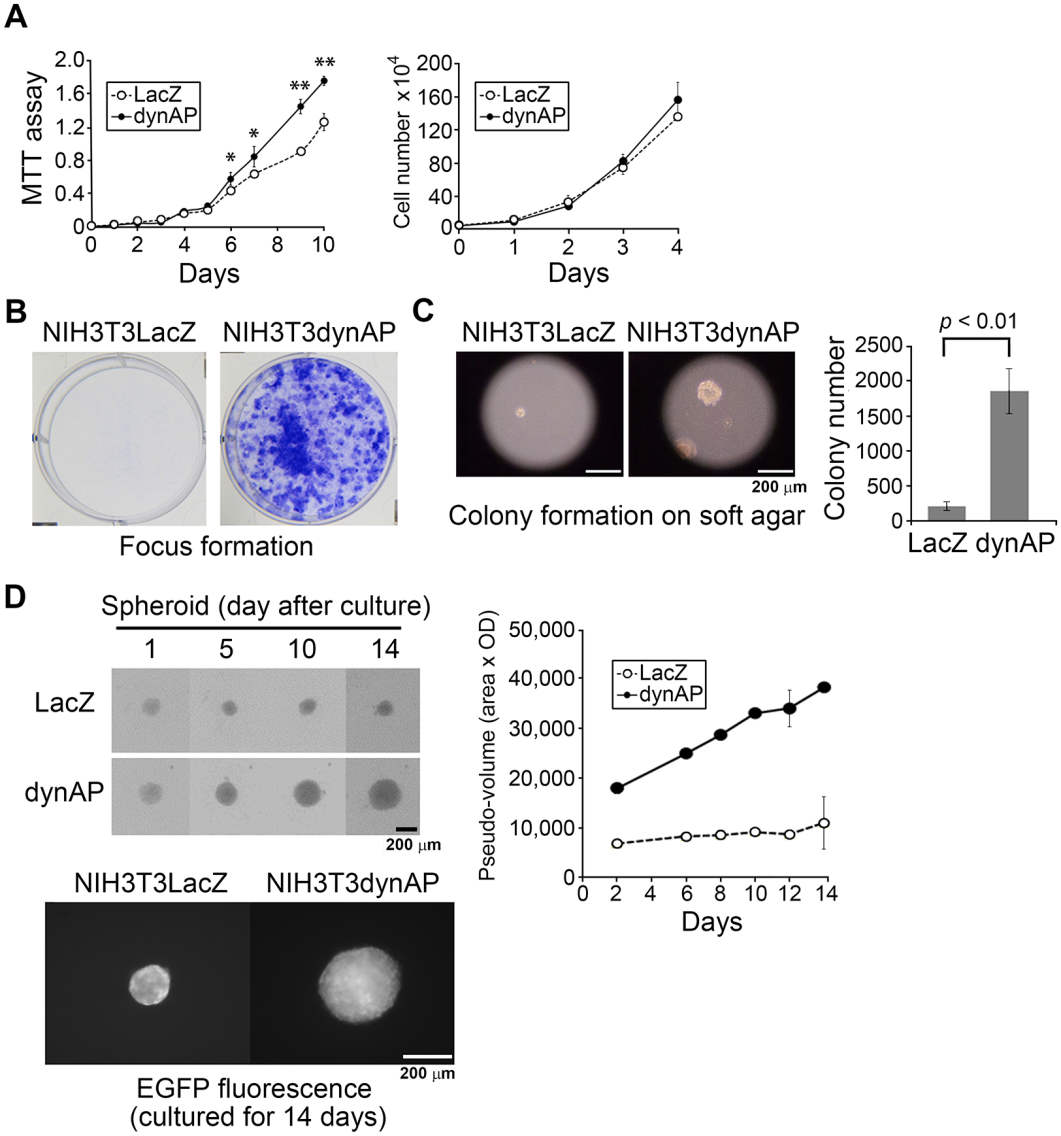
*In vitro* properties of NIH3T3 cells transformed by dynAP. (A) Proliferation in 2D culture. Cell proliferation was evaluated by the MTT method (left panel) and counting cell numbers (right panel). Error bars show the means ± SD (n = 4). NIH3T3dynAP vs NIH3T3LacZ cells; *p<0.05, p**<0.01. (B) Focus formation. The cells (5 × 10^4^/2 ml/well in 6-well plates) were cultured for 15 days and then stained with crystal violet to visualize foci. (C) Colony formation on soft agar. Colonies with diameters larger than 50 μm were counted on day 18 after plating 2 × 10^4^ cells/dish. Error bars show the means ± SD (n = 3). (D) Spheroid formation in 3D culture. Scanner images (left panels) indicate vigorous formation of spheroids by NIH3T3dynAP cells. Spheroid formation was quantified by measuring the pseudo-volume (area × optical density) (right panel). Error bars show the means ± SD (n = 3). NIH3T3dynAP vs NIH3T3LacZ cells; *p* < 0.01 at all points. Active spheroid growth of NIH3T3dynAP cells was also observed by fluorescence emitted from EGFP that was expressed in the parental NIH3T3 cells.

We compared cell proliferation of NIH3T3dynAP and NIH3T3LacZ cells in two-dimensional (2D) monolayer culture (Fig 2A). There were no substantial differences when cell numbers were counted. However, MTT assays showed low but statistically significant stimulation of cell proliferation in NIH3T3dynAP cells.

Despite the absence of a remarkable difference of cell proliferation in conventional 2D culture, NIH3T3dynAP cells showed hallmarks of *in vitro* cell transformation. NIH3T3dynAP cells produced a large number of foci that were derived from loss of contact inhibition of cell proliferation, whereas distinct foci were not found in NIH3T3LacZ cell cultures (Fig 2B). NIH3T3dynAP cells formed significantly more colonies with a larger size on soft agar in striking contrast to the control NIH3T3LacZ cells (Fig 2C). In addition, we compared spheroid formation of NIH3T3dynAP and NIH3T3LacZ cells in anchorage-deficient three-dimensional (3D) culture using ultralow attachment U-bottomed plates. NIH3T3dynAP cells formed a single, centrally located spheroid with a large size, whereas NIH3T3LacZ cells formed a small meager spheroid (Fig 2D). This finding was confirmed by measuring the fluorescence of EGFP that was expressed in the parental NIH3T3 cells. Measurement of the spheroid pseudo-volume (area × optical density) clearly showed robust growth of NIH3T3dynAP spheroids in a time-dependent manner, whereas NIH3T3LacZ spheroids grew at a minimal rate. These findings suggest that dynAP is likely oncogenic, prompting us to perform *in vivo* tumor formation assays.

Before tumor formation experiments, we prepared NIH3T3H-Ras cells from NIH3T3LacZ cells, which expressed constitutively active Ras (H-RasG12V). This mutant Ras shows strong *in vitro* cell transformation potency and *in vivo* tumorigenicity [12–15]. We also prepared NIH3T3H-RasdynAP cells by expressing dynAP in NIH3T3H-Ras cells. Thus, tumor formation by NIH3T3H-Ras and NIH3T3H-RasdynAP cells would validate our tumorigenic assay procedures and indicate whether dynAP cooperates with H-Ras in tumor formation. We characterized NIH3T3H-Ras and NIH3T3H-RasdynAP cells *in vitro* in comparison with NIH3T3dynAP cells. Both NIH3T3H-Ras and NIH3T3H-RasdynAP cells proliferated in 2D culture at much faster rates than NIH3T3dynAP cells and formed more colonies on soft agar with larger sizes (S1A and S1B Fig). Interestingly, NIH3T3H-RasdynAP cells formed larger colonies on soft agar than NIH3T3H-Ras cells and produced more colonies, albeit without statistical significance (S1B Fig). NIH3T3H-Ras cells formed spheroids with a larger size than those formed by NIH3T3dynAP cells (S1C Fig). Thus, the transformation potency of dynAP appeared to be weaker than that of the mutant Ras.

### Tumor formation of NIH3T3dynAP cells in nude mice

**Fig 3:**
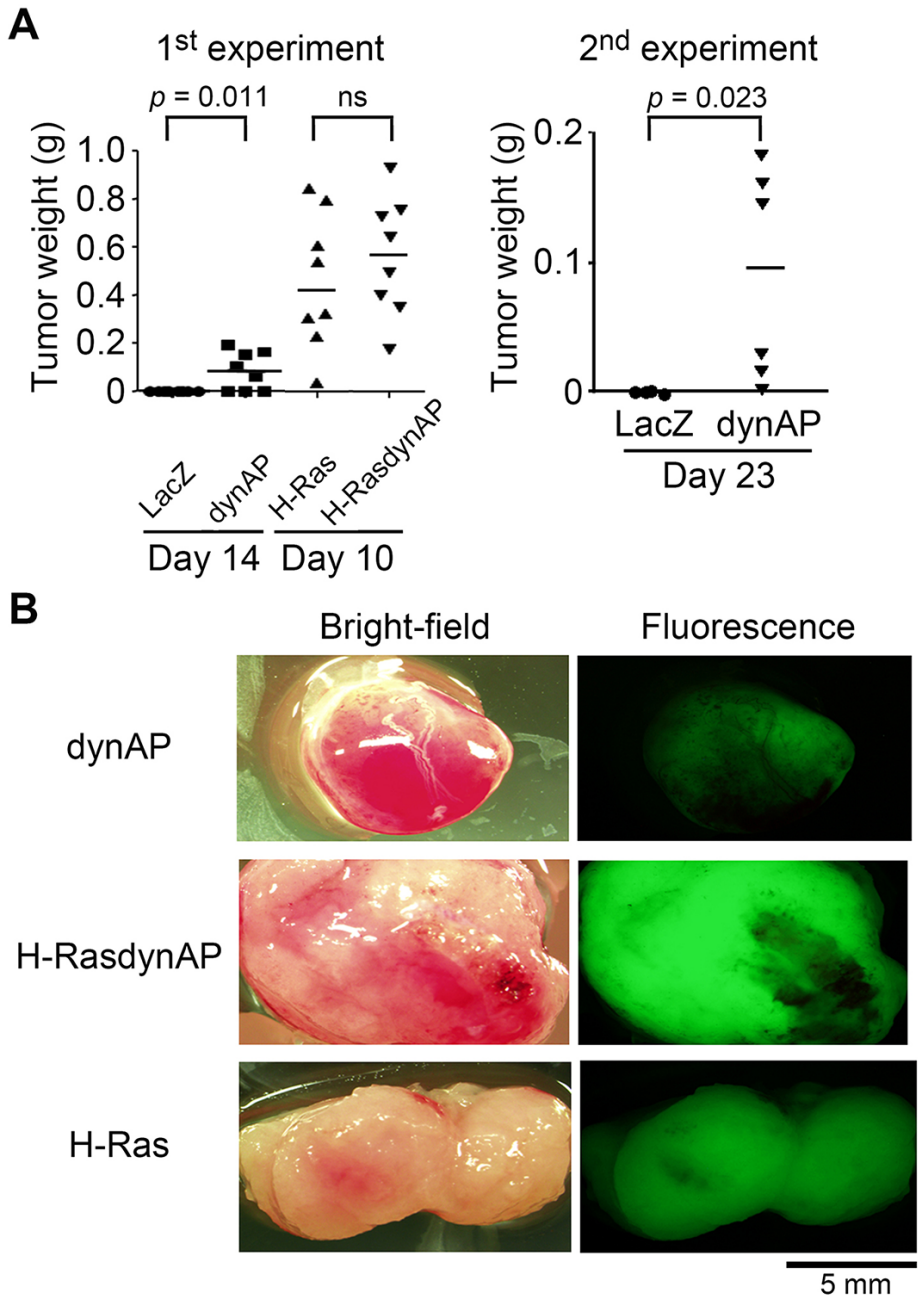
Tumor formation. (A) Tumor weights in nude mice that received NIH3T3 cells expressing dynAP, H-Ras, or H-RasdynAP. Tumor weights were measured at the indicated days after injection of the cells. Data were analyzed by the Mann-Whitney test. (B) Gross characteristics of tumors. Tumor tissues derived from NIH3T3dynAP and NIH3T3H-RasdynAP cells were highly vascularized compared with those derived from NIH3T3H-Ras cells.

Four NIH3T3 cells (NIH3T3LacZ, NIH3T3dynAP, NIH3T3H-Ras, and NIH3T3H-RasdynAP) were injected subcutaneously into nude mice. To evaluate animal health and suffering, symptom criteria, e.g. behavioral or movement abnormality, hyperalgesia, and decrease in food intake were monitored in every two days, and no abnormal symptom was observed. The gross appearances of the mice indicated the formation of large tumors in mice that received NIH3T3H-Ras and NIH3T3H-RasdynAP cells, whereas NIH3T3dynAP cells also formed distinct tumors but with smaller sizes than NIH3T3H-Ras- and NIH3T3H-RasdynAP cell-derived tumors (S2 Fig). Fig 3A shows the tumor weights. Five of eight mice that received NIH3T3dynAP cells formed tumors with an average weight of 84 mg. All mice that received Ras-expressing cells formed tumors with an average weight of 460 mg for NIH3T3H-Ras cells and 560 mg for NIH3T3H-RasdynAP cells. The average weight of NIH3T3H-RasdynAP cell-derived tumors was higher than that of NIH3T3H-Ras cell-derived tumors, but the difference was statistically insignificant. Tumors were undetectable in all eight mice that received NIH3T3LacZ cells. Because the tumors formed by NIH3T3dynAP cells were small and some (3/8) mice that received NIH3T3dynAP cells did not produce tumors in the first experiment, we performed a second experiment for confirmation. The results were similar to those of the first experiment. NIH3T3dynAP cells produced tumors in five of eight mice with an average weight of 92 mg, and tumors were undetectable in all four mice that received NIH3T3LacZ cells (Fig 3A, right panel).

Fig 3B shows representative tumors derived from NIH3T3dynAP, NIH3T3H-Ras, and NIH3T3H-RasdynAP cells. Fluorescence-positive areas indicate tumor tissues. Notably, compared with NIH3T3 cells expressing Ras alone (NIH3T3H-Ras), NIH3T3 cells expressing dynAP (NIH3T3dynAP and NIH3T3H-RasdynAP) formed tumors that were highly enriched with blood vessels. These vessels were clearly negative for fluorescence, indicating that they were derived from host cells and not NIH3T3 cells.

**Fig 4:**
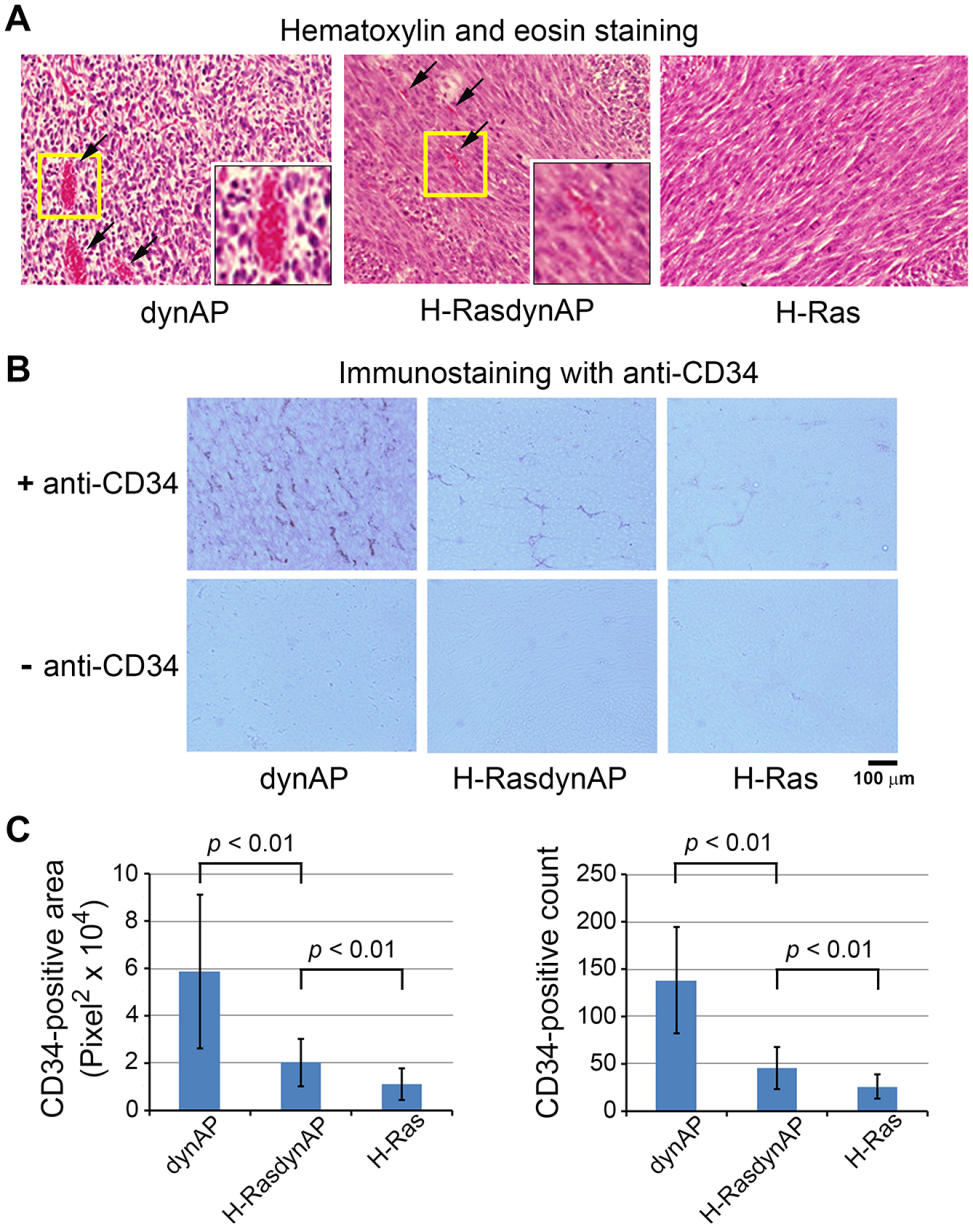
Analyses of tumor tissues. Tumor tissues were obtained from mice in the first experiment in Fig 3A. (A) Hematoxylin and eosin staining of tumors derived from NIH3T3dynAP, NIH3T3H-Ras, and NIH3T3H-RasdynAP cells. Arrows indicate capillaries filled with erythrocytes. Cell-cell adhesion appeared to be weak in NIH3T3dynAP cell-derived tumor tissue. (B) Sections stained with an antibody against CD34 (endothelial cell marker) and control sections stained without the antibody. The control sections were sections consecutive to those stained with the antibody. (C) Quantification of the CD34-positive area (left panel) and CD34-positive number (right panel). Data are the means ± SD (n = 9). Tumors obtained from 27 mice (nine mice for NIH3T3dynAP cells, nine for NIH3T3H-RasdynAP cells, and nine for NIH3T3H-Ras cells) were processed to prepare sections. One section of each tumor was randomly selected and two fields of each section were analysed.

Fig 4A shows hematoxylin and eosin-stained tumor tissues derived from NIH3T3dynAP, NIH3T3H-Ras, and NIH3T3H-RasdynAP cells. The NIH3T3dynAP cell-derived tissue contained many vessels filled with erythrocytes compared with the NIH3T3H-Ras cell-derived tissue. It also appeared that the NIH3T3H-RasdynAP cell-derived tissue contained more blood vessels than the Ras-expressing cell-derived tissue. Confirmation of these findings was performed by immunostaining of tumor sections with an antibody against CD34, an endothelial cell marker (Fig 4B), followed by quantification of the CD34-positive area or CD34-positive number (Fig 4C). Interestingly, the dynAP-expressing cell-derived tumor tissues showed the highest density of blood vessels and H-Ras repressed the angiogenic potency of dynAP, suggesting interplay between dynAP and H-Ras. These results indicate that dynAP stimulates angiogenesis in tumors.

In addition, the cell-cell contact in dynAP-expressing cell-derived tumor tissues appeared to be weaker than that in Ras-expressing cell-derived tissues (Fig 4A), which prompted us to examine E-cadherin levels as described later.

### Molecular mechanisms of dynAP-induced cell transformation

**Fig 5:**
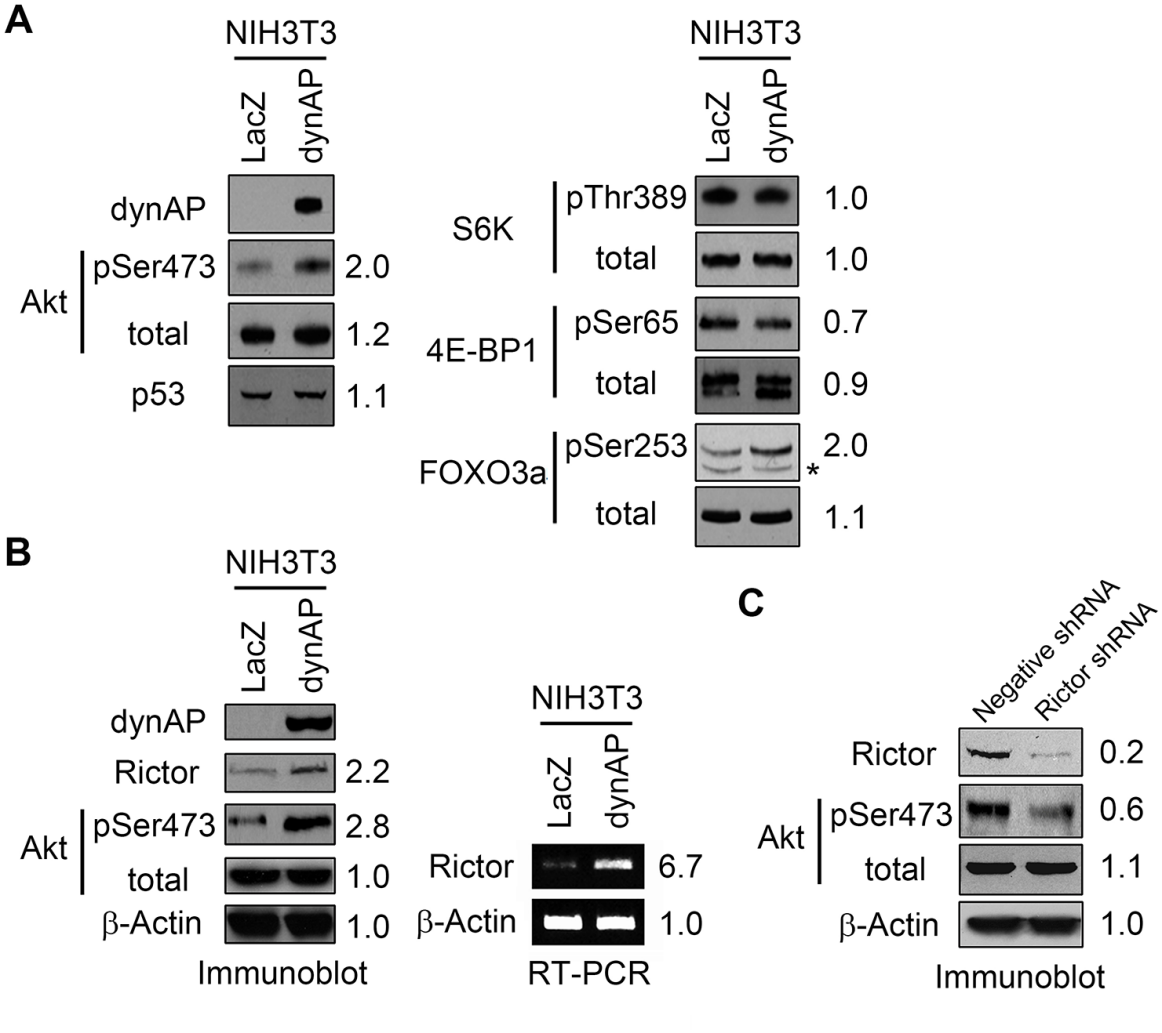
Molecular mechanisms of dynAP-induced transformation of NIH3T3 cells. (A) Immunoblots of key signaling molecules. Numbers on the right sides indicate relative band intensities of NIH3T3dynAP cells when those of NIH3T3LacZ cells were defined as 1. *non-specific band because its mobility differed from the FOXO3a band. (B) DynAP-induced increases of rictor protein and mRNA expression. β-Actin was used as a control. (C) Rictor knockdown in NIH3T3dynAP cells decreases AktSer473 phosphorylation. Apparent molecular masses of proteins on immunoblots were as follows. dynAP, 42 kDa; Akt, 56 kDa; p53, 53 kDa; S6K, 70 kDa; 4E-BP1, 18 kDa; FOXO3a, 97 kDa; rictor, 200 kDa, β-actin, 45 kDa.

We previously reported that overexpression of dynAP in HeLa cells promotes phosphorylation of AktSer473 [11]. In agreement with this finding, the same result was obtained by expression of dynAP in NIH3T3 cells (Fig 5A, left panel). We found no change in the level of p53 that has a strong effect on cell cycle progression and cell survival. Phosphorylation of S6K was also unchanged. A marginal decrease was seen in phosphorylation of 4E-BP1 upon dynAP expression, but it is unlikely that this decrease had a significant effect on cell proliferation because expression of dynAP did not change the rate of cell proliferation (see Fig 2A). Phosphorylation of FOXO3a was elevated upon dynAP expression (Fig 5A, right panel). Phosphorylation of this transcription factor inhibits nuclear translocation, resulting in promotion of cell survival. mTORC2 phosphorylates AktSer473, and phospho-AktSer473 recognizes FOXO3a as a substrate [9, 10]. Based on these facts, we hypothesized that dynAP might stimulate mTORC2 and focused on rictor, an indispensable subunit of mTORC2 to exert its activity towards AktSer473. We found that both the mRNA and protein levels of rictor were elevated upon dynAP expression in NIH3T3 cells (Fig 5B). To demonstrate the significance of the upregulation of rictor expression, we performed knockdown experiments. As shown in Fig 5C, knockdown of rictor reduced the phosphorylation of AktSer473.

**Fig 6:**
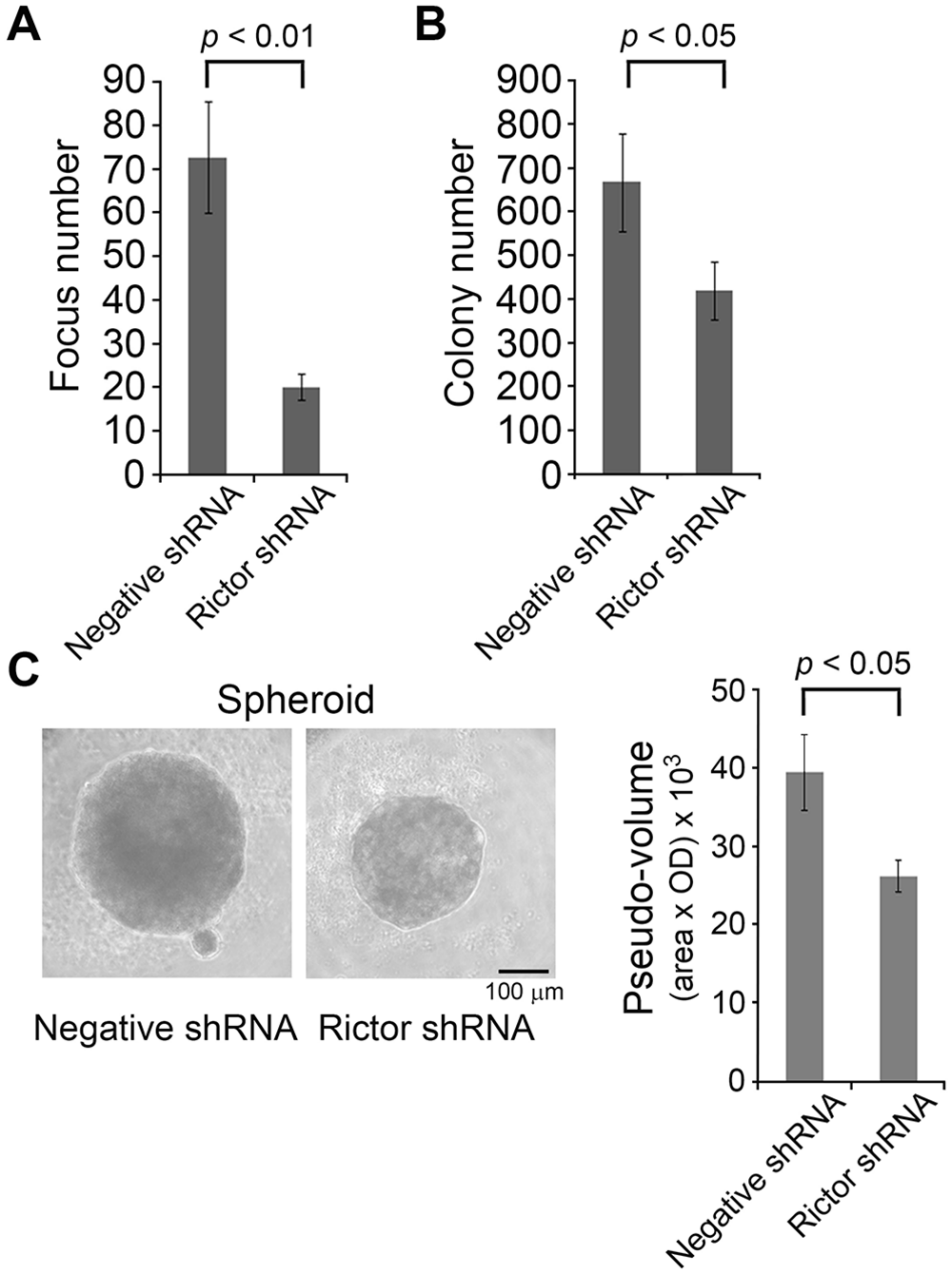
Rictor knockdown in NIH3T3dynAP cells represses dynAP-induced transformation. (A) Focus formation in 2D culture. (B) Colony formation on soft agar. (C) Spheroid formation in 3D culture. Left panel shows microscopic images of spheroids, and right panel shows quantification of spheroids. All data are the means ± SD (n = 3).

Furthermore, knockdown of rictor decreased the dynAP-induced *in vitro* transformation potency such as focus formation, colony formation on soft agar, and spheroid formation in 3D culture (Fig 6A–6C). Anoikis has been defined as cell death that occurs when anchorage-dependent cells are exposed to a matrix-deficient condition or an inappropriate matrix [16]. Resistance to anoikis is thought to be a hallmark of oncogenic transformation of cells. We confirmed that the poor spheroid formation of NIH3T3LacZ cells was due to apoptotic death in 3D culture, and that NIH3T3dynAP cells gained resistance to anoikis (S3 Fig). These results suggest a pivotal role of promoted activity of the mTORC2/AktSer473/FOXO3a pathway in dynAP-induced oncogenic transformation.

Histological observations of dynAP-expressing cell-derived tumors revealed two features in comparison with Ras-expressing cell-derived tumors (see Fig 4). DynAP-expressing cell-derived tumors contained many blood vessels and consisted of dispersed cells that suggested weak cell-cell contacts. The molecular mechanism of increased vascularization by dynAP is under investigation.

**Fig 7:**
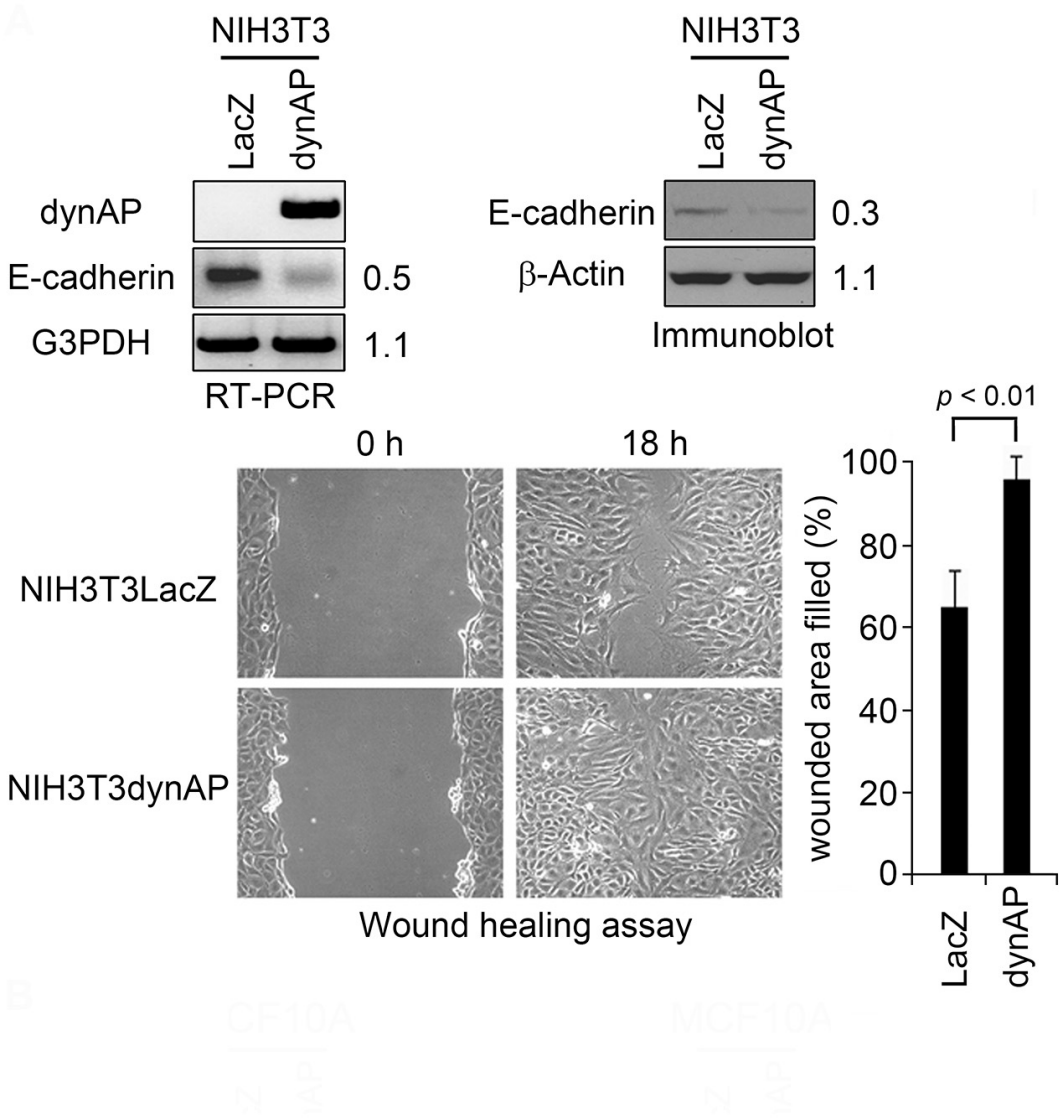
Expression of dynAP in NIH3T3 reduces E-cadherin expression and promotes cell motility. Cell motility was quantified by wound healing assays. Data are the means ± SD (n = 6).

We examined the involvement of E-cadherin in the formation of tumors with weak cell-cell contacts. E-cadherin mRNA levels were significantly decreased upon dynAP expression in NIH3T3 cells (Fig 7, left panel), which was accompanied by a reduction in E-cadherin protein expression (Fig 7, right panel). Consistent with this finding, wound-healing assays showed that cell migration was elevated upon dynAP expression (Fig 7, bottom panel). Thus, it is likely that the dynAP-induced downregulation of E-cadherin expression contributes at least partly to the weak cell-cell adhesion in NIH3T3dynAP cell-derived tumors.

## Discussion

We have previously undertaken yeast-based screening to identify novel functions of uncharacterized human proteins, and more than 100 proteins were found to inhibit the growth of yeast [17]. By extending this strategy to mutant yeasts that lacked a spindle checkpoint factor Mad2, we found that some of the human proteins inhibited the growth of mutant yeasts but not wild-type yeast. Among these proteins, an uncharacterized C18orf26-encoded protein has a transmembrane structure. Therefore, we characterized this protein with the expectation that it might function as a receptor of environmental cues such as growth factors and extracellular matrix proteins. Although our initial hypothesis has not been proven, this protein, referred to as dynAP because of its physical interaction with dynactin in a microtubule complex, has been found to stimulate AktSer473 phosphorylation [11]. As shown in the present study, further characterization has uncovered remarkable features of dynAP. Specifically, we demonstrated that expression of dynAP in mouse cells induces oncogenic transformation. Although tumor formation in a model animal system is not necessarily an indication of tumorigenicity in humans, our findings warrant further studies of dynAP as a potential oncoprotein.

Akt is phosphorylated at two sites, Thr308 and Ser473 [18]. PDK1 is responsible for phosphorylation of AktThr308. Phosphorylation of Thr308 is a prerequisite and sufficient for the full action of the Akt-mTORC1 pathway [1]. Phosphorylation of AktSer473 produces the fully active kinase with the altered substrate specificity. The identity of the AktSer473 kinase was elusive for long time, but it has been revealed that mTORC2 [7, 9–12, 19] and DNA-PK [8] phosphorylate AktSer473. Expression of dynAP in NIH3T3 cells elevated the expression of the rictor subunit at both mRNA and protein levels. Rictor is essential for mTORC2, but it is not involved in mTORC1 [6]. Knockdown of rictor in NIH3T3dynAP cells abrogated dynAP-induced elevation of AktSer473 phosphorylation and concomitantly reduced the *in vitro* transformation potency of dynAP. The mechanism by which rictor mRNA is induced by dynAP remains to be investigated.

Akt phosphorylated at Ser473 phosphorylates FOXO3a at multiple sites, including Ser253 and Thr32, and prevents nuclear translocation of FOXO3a, inhibiting its function as a transcription factor of pro-apoptotic genes [9, 10, 20, 21]. Thus, phosphorylation of FOXO3a by the AktSer473 phosphoenzyme supports the survival of cells receiving apoptotic stimuli. Phosphorylation of FOXO3a at Ser253 was stimulated upon expression of dynAP in NIH3T3 cells. In addition, dynAP conferred resistance to apoptosis in 3D-cultured cells. Taken together, our results indicate that activation of the mTORC2-AktSer473-FOXO3a pathway contributes to dynAP-induced oncogenic transformation.

Although considerably less is known compared with mTORC1, there are increasing indications that mTORC2 is involved in human cancer [6, 22–28]. Stimulation of the anti-apoptotic pathway (AktSer473-FOXO3a) via mTORC2 likely plays a critical role in dynAP-induced cell transformation, but it is premature to propose that this stimulation is entirely responsible for the tumorigenic potency of dynAP. Reorganization of the cellular cytoskeleton, such as actin filaments, may be needed to provide a growth advantage to NIH3T3dynAP cells in anchorage-deficient culture. In this context, it is interesting that reports have implicated mTORC2 in the regulation of actin organization through PKCa [22, 29] or Rho-GTPases [30].

mTORC1 is one of the downstream effectors of Akt, while mTORC2 is upstream of Akt. The factors upstream of mTORC2 are not well understood. Ribosomes have been shown to activate mTORC2 [31, 32]. Tumor-suppressive microRNAs exert their function by repression of mTORC2 activity through a reduction in rictor expression [27, 28]. It appears that dynAP is a novel modulator that functions upstream of mTORC2, but the mechanism remains to be studied.

It is noteworthy that NIH3T3dynAP cells generated tumors with two characteristics, abundant blood vessels and weak cell-cell adhesion. Although the mechanism of dynAP-induced vascularization remains unknown, the weak cell-cell adhesion may be explained by the reduced expression of E-cadherin. Epithelial-to-mesenchymal transition (EMT) occurs during embryonic development, but it has been implicated in promotion of cancer invasion and metastasis [33, 34]. E-cadherin is a key molecule that represses EMT by strengthening cell-cell interactions. Therefore, the dynAP-induced reduction in E-cadherin levels may give rise to highly metastatic tumors.

In conclusion, dynAP may be a new target for cancer therapy because it initiates oncogenic cell transformation and facilitates tumor malignancy. Arguably, our findings raise more fundamental questions regarding dynAP, such as its physiological function, expression in human normal and cancerous tissues, and regulation of expression.

## Supporting Information

S1 Fig Comparison of the growth properties of NIH3T3dynAP, NIH3T3H-Ras, and NIH3T3H-RasdynAP cells. (A) Growth in 2D culture. Cells were seeded in a 96-well plates (300 cells/well) and cultured for the indicated periods. Cell growth was measured by MTT assays. Error bars show the means ± SD (n = 4). (B) Colony formation on soft agar. Cells were seeded in 6-cm dishes (2 × 10^4^ cells/dish) and cultured. Left panel shows colonies on day 6, and right panel shows colony numbers counted on day 14. Error bars show the means ± SD (n = 3). ns, not significant. (C) Spheroid formation in 3D culture. Cells were seeded (500 cells/well) in an ultralow cell adhesion plate and cultured for 4 days. Magnification, ×100.
(TIF)

S2 Fig Tumors in mice injected with NIH3T3LacZ, NIH3T3dynAP, NIH3T3H-Ras, and NIH3T3H-RasdynAP cells. Photographs were taken on day 14 after injection of NIH3T3LacZ and NIH3T3dynAP cells and on day 10 after injection of NIH3T3H-Ras and NIH3T3H-RasdynAP cells (see the first *in vivo* experiment in Fig 3A). Red circles indicate tumors.
(TIF)

S3 Fig DynAP represses apoptosis in 3D culture. NIH3T3LacZ and NIH3T3dynAP cells were seeded in ultralow cell adhesion plates (Sumilon PrimeSurface 35 mm-dish from Sumitomo Bakelite, Tokyo, Japan) at a density of 2 × 10^5^ cells per dish and cultured for 4 days. Gently trypsinized cells were analyzed by flow cytometry to estimate apoptotic cells in 3D culture. The percentages of apoptotic cells (sub-G1 fractions) are indicated. To clearly show sub-G1 fractions, the x-axis is a logarithmic scale.
(TIF)
