# Supplementary figures and images for: Human Dynactin-Associated Protein Transforms NIH3T3 Cells to Generate Highly Vascularized Tumors with Weak Cell-Cell Interaction
Source: PLoS One. 2015 Aug 18;10(8):e0135836. doi: 10.1371/journal.pone.0135836 (PMC4540312; doi:10.1371/journal.pone.0135836)

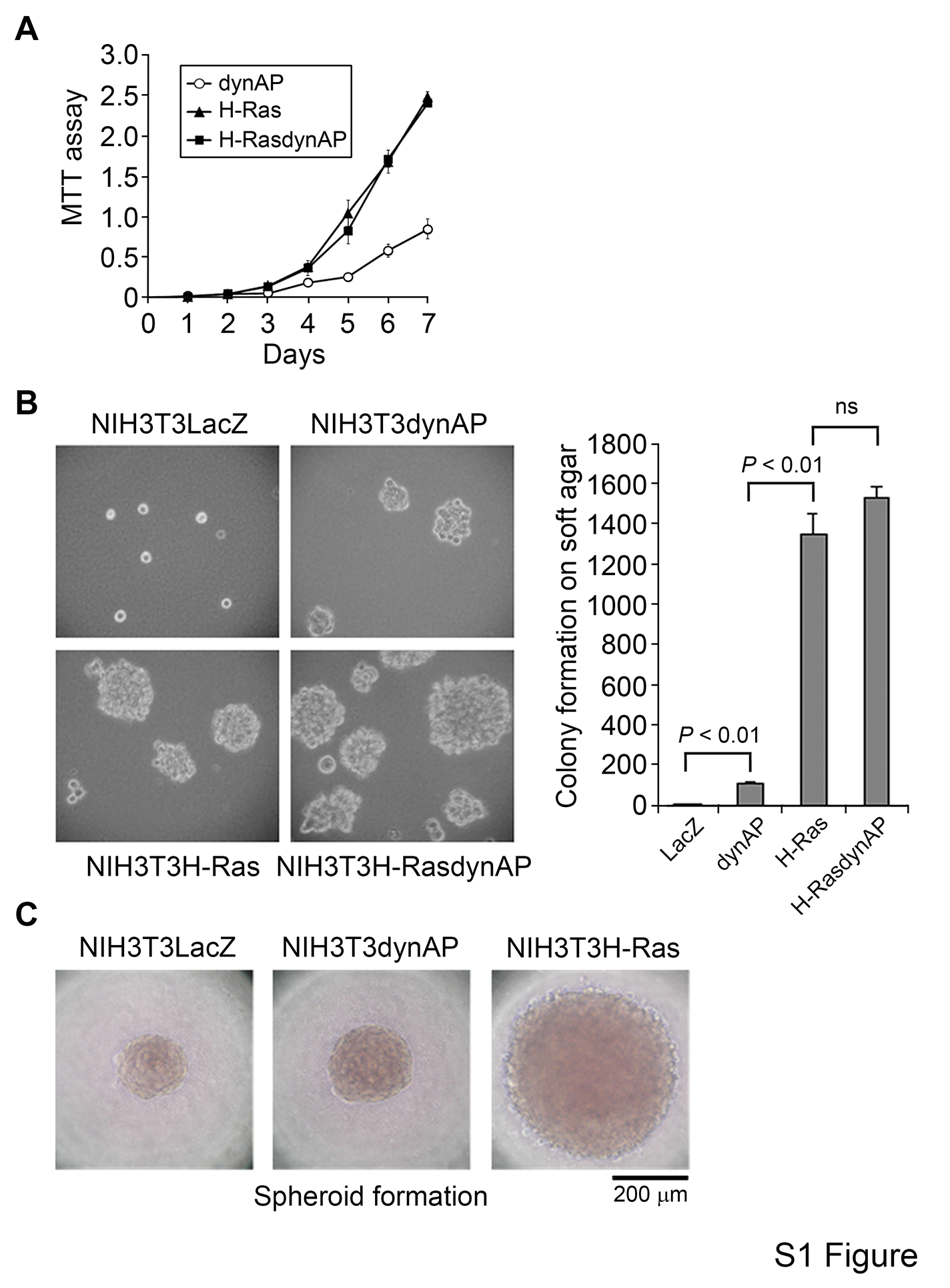

Supplement: S1 Fig — (A) Growth in 2D culture. Cells were seeded in a 96-well plates (300 cells/well) and cultured for the indicated periods. Cell growth was measured by MTT assays. Error bars show the means ± SD (n = 4). (B) Colony formation on soft agar. Cells were seeded in 6-cm dishes (2 × 10^4^ cells/dish) and cultured. Left panel shows colonies on day 6, and right panel shows colony numbers counted on day 14. Error bars show the means ± SD (n = 3). ns, not significant. (C) Spheroid formation in 3D culture. Cells were seeded (500 cells/well) in an ultralow cell adhesion plate and cultured for 4 days. Magnification, ×100. (TIF) [file pone.0135836.s001.tif]

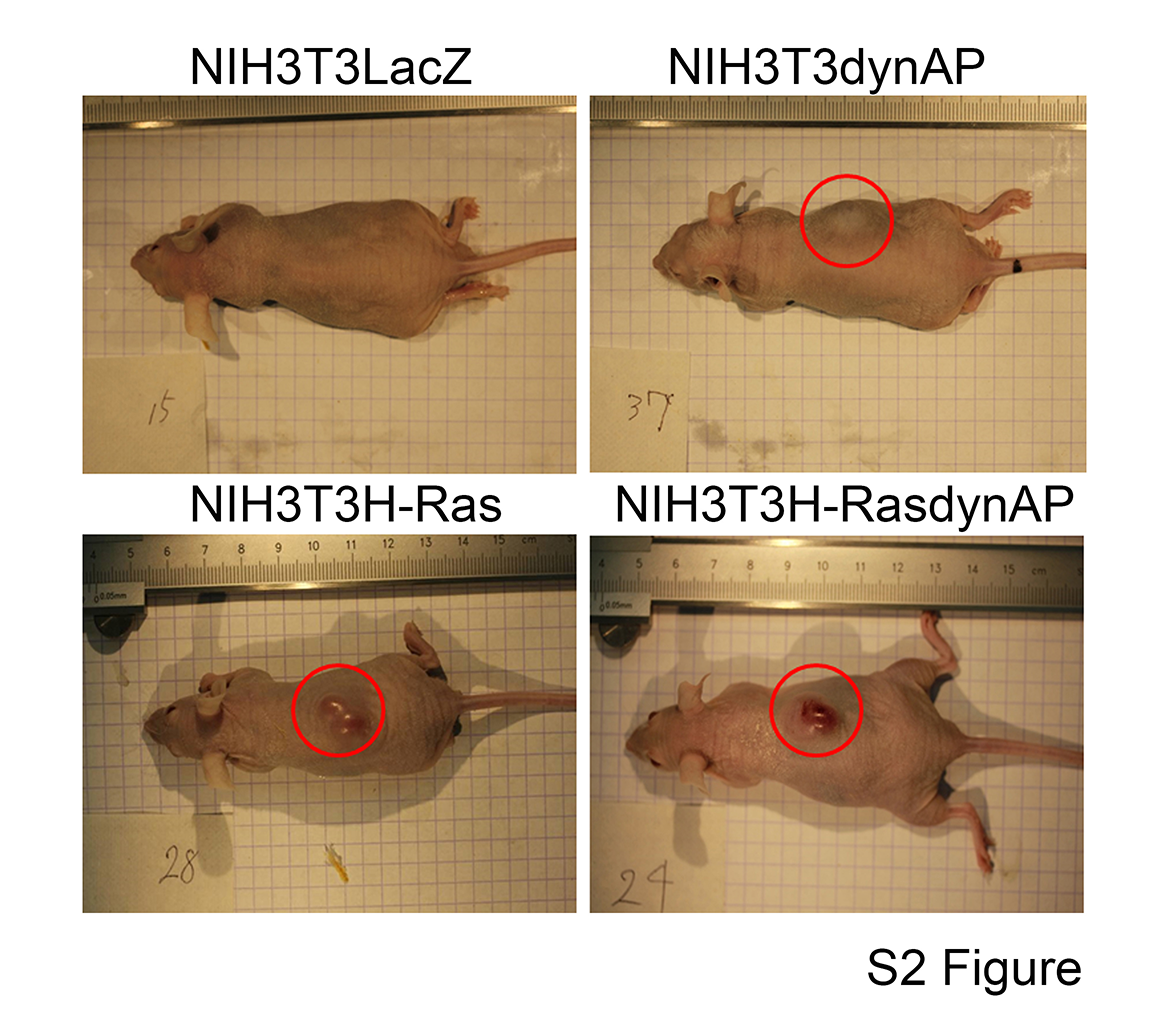

Supplement: S2 Fig — Photographs were taken on day 14 after injection of NIH3T3LacZ and NIH3T3dynAP cells and on day 10 after injection of NIH3T3H-Ras and NIH3T3H-RasdynAP cells (see the first in vivo experiment in Fig 3A). Red circles indicate tumors. (TIF) [file pone.0135836.s002.tif]

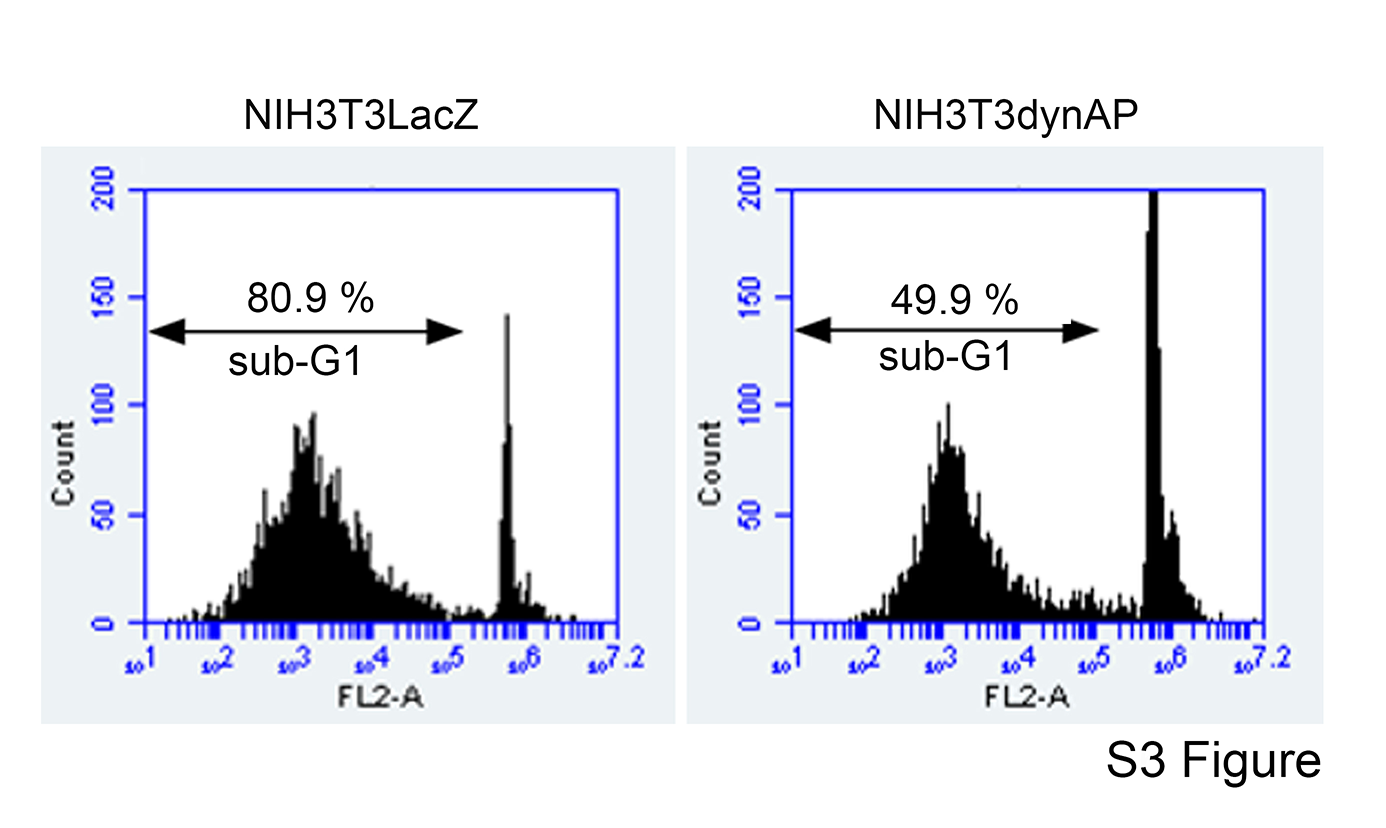

Supplement: S3 Fig — NIH3T3LacZ and NIH3T3dynAP cells were seeded in ultralow cell adhesion plates (Sumilon PrimeSurface 35 mm-dish from Sumitomo Bakelite, Tokyo, Japan) at a density of 2 × 10^5^ cells per dish and cultured for 4 days. Gently trypsinized cells were analyzed by flow cytometry to estimate apoptotic cells in 3D culture. The percentages of apoptotic cells (sub-G1 fractions) are indicated. To clearly show sub-G1 fractions, the x-axis is a logarithmic scale. (TIF) [file pone.0135836.s003.tif]
